# Acute gastroenteritis—changes to the recommended original oral rehydrating salts: a review

**DOI:** 10.3389/fped.2023.1294490

**Published:** 2023-12-18

**Authors:** Carlos Lifschitz, Oleksii Kozhevnikov, Christine Oesterling, Amira Anbar, Steven Walker

**Affiliations:** ^1^ Hospital Italiano de Buenos Aires, Buenos Aires, Argentina; ^2^ MedibiotiX GmbH, Baden-Baden, Germany; ^3^ Eastmead Surgery, Greenford, and Imperial College London, London, United Kingdom; ^4^ St. Gilesmedical, London, United Kingdom; ^5^ St. Gilesmedical (London & Berlin) & University of Applied Sciences, Bremen, Germany

**Keywords:** review, acute gastroenteritis (AGE), oral rehydrating solution, probiotics and prebiotics, zinc, low osmolar oral rehydration solution, rotavirus diarrhea

## Abstract

The World Health Organization recommended a formulation of oral rehydration salts as the intervention of choice for the treatment of acute gastroenteritis. While of value for the replacement of fluids and electrolytes, the formulation does not reduce stool volume, frequency, or symptom duration. This may prevent wide acceptance. To increase tolerability, shorten the duration of diarrhea and improve parental quality of life, several modifications to the original formula have been proposed. These include; low osmolarity, rice-based, glucose polymers as an alternative to glucose, the addition of probiotics, prebiotics and/or zinc, and various other ingredients. Here we summarize evidence regarding such changes and additions.

## Introduction

Acute gastroenteritis (AGE) is a common clinical problem in children. Acute diarrheal disease accounts for 179 million outpatient visits every year in the USA (1). AGE is a largely self-limited disease, typically due to a viral intestinal infection. Common organisms are rotavirus and norovirus, followed by adenovirus and astrovirus. Signs and symptoms include watery diarrhea, stomach cramps, nausea, vomiting, and sometimes fever. AGE is a leading cause of morbidity and mortality, particularly in developing countries (2). By comparison, mortality from AGE is low in Western countries, while morbidity is often high, particularly among infants. Children who attend daycare have almost twice the risk of AGE compared to those who are home-cared (3). Moreover, infected children may spread AGE to household members. This occurs approximately once in every three AGE episodes (3), resulting in a 3- to 8-fold increased risk for secondary AGE in young children (4). Studies from developed countries indicate that the monthly community incidence of AGE is 2.6%–11.1%, corresponding to 0.3–1.5 episodes/person-year (5). Children with disabilities are at higher risk of AGE.

For more than a quarter of a century, the World Health Organization (WHO) and the United Nations Children's Fund (UNICEF) advocated the use of a single glucose-based Oral Rehydration Salts (ORS) formula, comprising of 90 mEq/L of sodium with an osmolarity of 311 mOsm/L to prevent or treat mild to moderate diarrhea-induced dehydration. This recommendation was irrespective of patient age or causation (6). While safe and of proven benefit, this product alone does not reduce stool volume, frequency of defecation or symptom duration (7). This has prevented universal applicability.

In 1978, an editorial in the Lancet stated “The discovery that sodium transport and glucose transport are coupled in the small intestine, so that glucose accelerates absorption of solute and water, is potentially the most important medical advance of this century” (8). In other words, the presence of sodium and potassium in ORS, serves to replace intestinal losses, while glucose stimulates small bowel absorption of sodium.

Despite rotavirus vaccination, the morbidity from childhood AGE in developed countries remains high. This can have a significant societal impact. Effects include; increased medical expenditure, the need for alternative care (e.g., babysitting), and loss of productivity. The latter is especially an issue among dual-income and single-parent families (5). A survey in three European countries concluded that families with a child affected by AGE, experience stress and anxiety which in turn, interferes with daily-life tasks and work (9). Modern medicine increasingly emphasizes the importance of considering how illness more widely affects both the patient and their family. No longer are clinical improvement and safety of sole interest. Consequently, tools such as the health-related (HR) Quality of Life (QoL) indicator have become important outcome measures for new therapies. One way to improve HRQoL scores is to minimize the severity and duration of the disease. In respect of AGE, modifying the ORS by the addition of ingredients to shorten symptom duration, could result in an improved HRQoL, and reduce the financial burden. To that end, numerous ingredients have been tested to optimize and improve ORS treatment (i-ORS; Table 1). This review provides an update of potentially beneficial modifications for treating non-cholera AGE.

**Table 1:** Summary of selected principal modifications to wHO-ORS composition and ingredients.

Type of modification	Specifics	Effects	Reference
Decrease osmolarity	Lower sodium	• Lower stool volume • Less vomiting	Hahn S. et al. (10)
	Rice-based	• No benefit • Some children experienced increased stool volume/ loss	Guiraldes E et al. (11)
	Glucose polymers	• Lower stool volume • Shorter duration of diarrhea • No difference in vomiting	Gregorio GV. et al. (12)
Addition of amino acids	Alanine glutamine	No benefit	Ribeiro Júnior HDC. et al. (13) Gutierrez C. et al. (14)
Supplementation with Zinc (Zn)		Shorter duration of diarrhea (mean 12-hrs) Fewer children with persistent diarrhea until Day 7	Lazzerini M. et al. (15)
		Lower dose Zn (5 and 10 mg/day) results in a similar reduction in diarrhea compared to standard recommended dose but less vomiting	Dhingra U. et al. (16).
		Adding Zn to ORS reduced risk of severe diarrhea	Sazawal S. et al. (17) Brown KH. et al. (18) Bishai D. et al. (19) Gebremedhin S. et al. (20)
		Adding Zn is cost effective	Mejia A. et al. (21)
Addition of probiotics	*Lactobacillus (Lacticaseibacillus) rhamnosus* (LGG)	Reduction in the duration of diarrhea	Vlasova AN. et al. (22) McFarland LV. et al. (23). Steyer A. et al. (24).
	*Saccharomyces cerevisiae boulardii* (SB)	Reduction in the duration of diarrhea	Vlasova AN. et al. (22) McFarland LV. et al. (23) Steyer A. et al. (24)
	*Limosilactobacillus reuteri* DSM 17938 (LR)	Reduction in the duration of diarrhea	Peng Y. et al. (1) Szajewska H. et al. (25). Shornikova AV. et al. (26) Augustina R. et al. (27) Francavilla R. et al. (28) Dinleyici EC. et al. (29) Gutierrez-Castrellon *P* et al. (30) Dinleyici EC. et al. (31) Maragkoudaki M. et al. (32)
	*B.claussii*	Reduction in diarrhea duration found among Indian children	McFarland LV. et al. (23)
Addition of mixed probiotics	*SB + Baccilus subtilis* combination	Early administration (<48 h) can shorten diarrhea	Ghosh A. et al. (33)
	VSL#3 mixture	Improved recover rates	Dubey AP. et al. (34).
Addition of prebiotics	Oral rehydration salt (ORS) 220 mOsm/L. vs. ORS 200 mOsm/L + Zn + fructo-oligosaccharide + xylo-oligosaccharides	25% increase symptom resolution at 72-hours	Passariello A. et al. (35)
Addition of symbiotics	e.g. probiotical	Shorter duration of diarrhea and hospitalization and severity	Yang B. et al. (36) Gonzales Ochoa G. et al. (37) Vandenplas Y. et al. (38) Kluijfhout S. et al. (39) Hojsak I. et al. (40)

## Proposed oral rehydration salts modifications

### Reduced osmolarity

In Western countries, the use of ORS solution with osmolarity lower than that of plasma has been recommended to avoid the risk of hypernatremia. The clinical efficacy of reduced-osmolarity ORS was compared to that of standard ORS in children with AGE, in four developing countries. By reducing the solution's glucose and salt concentrations, the revised version had an osmolarity of 224 mmol/L. A multicenter trial conducted almost 30-years ago found that stool output was 39% lower, requirement for ORS replacement 18% less, and duration of diarrhea shortened by 22%, compared to conventional ORS (41). Modern ORS products generally have osmolarities in the range of 210–260 mmol/L. A 2002 Cochrane review compared reduced osmolarity ORS with WHO standard ORS in children admitted to the hospital with AGE (10). It was concluded that reduced osmolarity ORS, when compared to WHO standard ORS, was associated with fewer unscheduled intravenous fluid infusions, lower stool volume, and less vomiting. No additional risk of developing hyponatremia when compared with WHO standard ORS was detected.

### Formulations using rice-based oral rehydration salts

In a study looking at the treatment of dehydrated infants with watery, non-cholera diarrhea Guiraldes et al., found that a commercial ORS formula containing rice powder was non-superior to standard WHO/UNICEF-advocated glucose-ORS (11). Indeed, in some children, the trial preparation increased stool losses.

### Polymer-based oral rehydration salts

Glucose polymers (GP), derived from cornstarch or rice, are another option to reduce ORS osmolarity (7). How results compare with reduced osmolarity glucose-based ORS (osmolarity ≤270) was examined in a 2016 Cochrane review. Eight relevant data sources (752 participants) were examined: seven employed rice as the GP source and one trial used amylase resistant starch (12). Two trials were in adults and the remainder in children. Only one study (99 participants; low quality evidence) assessed mean difference (MD) in stool volume in the first 24 h; this was lower in the polymer-based ORS (−24.60 ml/kg, 95% CI −40.69 to −8.51).

Across five trials (364 participants; low quality evidence), the mean duration of diarrhea was shorter following administration of a polymer ORS by about 8 h (MD −8.24 h, 95% CI −13.17 to −3.30). There was, however, much variability between results (range: 3 to 13 h *I* statistic = 86%). Four trials (376 participants; very low quality evidence) found no difference in the risk of unscheduled intravenous fluid administration (RR 0.66, 95% CI 0.43–1.02; *I* statistic = 30%), vomiting (very low-quality evidence), and hyponatremia (very low-quality evidence) (12).

### Addition of amino acids

Certain neutral amino acids (e.g., glycine, L-alanine, L-glutamine) can enhance the absorption of sodium ions and water from the gut. Whether this is of clinical benefit has undergone limited study. In one trial involving 20 male infants <1-year with acute diarrhea, the addition of 30 mmol/L alanine to the standard WHO-ORS resulted in no additional improvement compared with those fed standard WHO-ORS (13). A similar conclusion was reached in a Mexican study looking at acute non-cholera diarrhea. This double-blind, randomized controlled trial (RCT) included 147 participants aged 1–60 months with mild to moderate dehydration. Participants were assigned to receive either WHO-ORS (74 children), or L-glutamine ORS (73 children). Follow-up was continued until successful rehydration (14). The authors found that adding L-glutamine did not offer clear clinical benefit compared to standard WHO-ORS. More data may emerge from an ongoing study comparing the efficacy of amino acid-based ORS “VS002A” with standard glucose-based WHO-ORS in a large cohort of infants and young children with acute non-cholera watery diarrhea (42).

### Zinc

Zinc (Zn) is a trace mineral, with a concentration in the human body second to that of iron (43). Zn is ubiquitous within cellular components, in contrast to iron which is distributed throughout the body. Both have defined physiological roles. Data primarily from in-vitro studies using intestinal cell lines and animal models, demonstrate that Zn influences gene expression and oxidative stress (44, 45). It modulates the immune response and resistance to infection, supports the intestinal barrier against noxious substances, stimulates regeneration of the epithelium and brush border, and may aid the clearance of pathogens (44, 45). These effects reduce the duration, severity, and risk of diarrheal disease via improved absorption of water and electrolytes (45).

The potential benefits of Zn in AGE were examined in a Cochrane review of thirty-three trials in diverse populations involving 10,841 children (15). For infants older than 6-months, Zn supplementation may shorten the average duration of diarrhea by around half a day and probably reduces the number of children whose diarrhea persists until day 7. Conversely, in children under 6-months, the evidence suggests that Zn supplementation has no effect on the mean duration of diarrhea or the number who still have diarrhea at the same time point. In children with persistent diarrhea (defined as diarrhea lasting more than 7-days), Zn supplementation probably shortens the average duration by around 16-hours. The authors concluded that in areas where the prevalence of Zn deficiency or malnutrition is high, supplementation may be of benefit in children over 6-months of age. None of the included trials reported serious adverse events.

Zn supplementation during diarrheal illnesses is recommended in developing countries where deficiencies may exist by the World Health Organization (WHO) and the United Nations Children's Fund (UNICEF). This is based on several trials demonstrating the beneficial effects of Zn (46). Here, the suggested dosage is 10 mg daily for infants under 6-months and 20 mg for older children. Effectiveness of this regimen was confirmed in a Swiss study (47).

Unfortunately, one potential side effect of Zn administration is vomiting. This may reduce compliance. The issue was addressed in a large study by Dhingra et al. (16). The researchers randomized 4,500 Indian and Tanzanian children aged 6 to 59 months with acute diarrhea to receive 5 mg, 10 mg, or 20 mg of zinc sulfate for 14 days. The percentage of children with diarrhea >5 days was 6.5% in the 20 mg group, 7.7% in the 10 mg group, and 7.2% in the 5 mg group. The differences between 20 mg and 10 mg (1.2%; upper bound of one-sided 98.75% CI 3.6%) and between 20 mg and 5 mg dosages (0.7%; upper bound 3.0%) were both below the 4% noninferiority margin. Similarly, the mean number of loose stools were 10.7, 10.9, and 10.8 in the 20 mg, 10 mg and 5 mg groups, respectively. Again, the differences were both below the noninferiority margin of 2 stools: 20 mg vs 10 mg groups 0.3 (upper bound 1.1), and 20 mg vs. 5 mg group 0.1 (upper bound 0.9).

Regarding early vomiting (first 30 min after Zn administration) this occurred in 19.3% after 20 mg, 15.6% after 10 mg, and 13.7% after 5 mg. The frequency of early vomiting was significantly lower in the 10 mg (relative risk 0.81, 97.5% CI 0.67–0.96) and 5 mg (0.71, 97.5% CI 0.59–0.86) groups. Both these dosages also reduced vomiting after 30 min. It is concluded that lower doses of Zn (5 mg and 10 mg) are non-inferior for the treatment of diarrhea in children; they are better tolerated (less vomiting) than the standard 20 mg dose. These findings are supported by a trial from Australia which showed that even small doses of Zn (3 mg) can improve intestinal permeability, though may not be sufficient to influence recovery (48).

Sazawal et al. proposed that mixing Zn with ORS is likely to be of added value as Zn supplementation exhibited more than 20% risk reduction in severe diarrhea (17). Several articles from sub-Saharan Africa, South Africa, Myanmar, Ethiopia, and Colombia, among others, found that the addition of Zn to ORS was cost-effective (18–20). In a Colombian study from 2015, Mejia et al. determined the cost-effectiveness of Zn supplementation for the treatment of AGE in children younger than 5 years (21). Results from their model indicate that Zn supplementation is a dominant strategy; it is less costly and more effective than standard treatment without Zn (reduction of $ 8.14 USD per child). The authors concluded that Zn for the treatment of AGE is a highly cost-effective strategy and is recommended for inclusion in the benefit plan of the Colombian health system. It should be noted that his intervention is sensitive to changes in predicted severity, i.e., it is more cost-effective in children with a higher risk of persistent diarrhea and hospitalization. In contrast to the above, one study from India did not find that Zn and copper supplementation were cost saving (49).

### Probiotics

Probiotics are live microorganisms that, when administered in adequate amounts, confer a health benefit on the host (50). Species of *Lactobacillus* and *Bifidobacterium,* and the yeast *Saccharomyces boulardii* (SB), are the most employed probiotics for diarrhea (22, 50). Probiotics can help to maintain homeostasis and balance among different species of intestinal microorganisms. They can also have a role in activating the immune system and regulating immune responses. The antiviral effects of probiotics are proven. These include, direct modulation of chemical, microbial, physical, and immune barriers, or via probiotic metabolites, and host signaling pathways (51).

Both *Lactobacillus* and *Bifidobacterium* species have been linked to the production of the cytokines interleukin (IL)25, IL33, and transforming growth factor (TGF) by intestinal cells. Similarly, there is an association between the production of IL22, by innate immune cells and IL10, IL12, IL25, and TGF, by antigen-presenting cells. Potential benefits include improved intestinal barrier function, reduced effector, and increased regulatory immune responses (22). Regarding AGE, probiotics including *Lactobacillus* (now *Lacticaseibacillu*s) *rhamnosus* GG (LGG), SB, and also *Limosilactobacillus reuteri* DSM 17938, formally known as *Lactobacillus reuteri* (LR), are effective in reducing the severity and duration of symptoms. The European Society for Paediatric Gastroenterology, Hepatology and Nutrition (ESPGHAN) published recommendations for the use of probiotics in AGE (25). For a reduction in the duration of diarrhea, length of hospitalization, and stool output, ESPGHAN considers there is evidence to support:

1) LGG, at a dose of ≥10^10^ colony forming units (CFU)/day, for 5–7 days.

For a reduction in the duration of diarrhea, there is evidence in support of:

2) SB, at a dose of 250–750 mg/day, for 5–7 days.
3) LR, at daily doses 1 × 10^8^ to 4 × 10^8^ CFU, for 5 days.
4) A combination of LGG 19070-2 and LR (both at a dose of 2 × 10^10^ CFU) for 5 days.

Conversely, the combination of *Lactobacillus helveticus* R0052 and *L rhamnosus* R0011 cannot be recommended as there is no evidence to show a beneficial effect.

Much clinical data is now available concerning the effectiveness of different probiotics, notably LR, LGG and SB. Originally isolated from the vagina of a healthy woman. LT is the most well documented. This follows Shornikova et al. who were the first to demonstrate a dosage-dependent shortening of acute watery diarrhea duration in children (26). Since then, several RCTs have shown that LR can reduce the frequency, duration, and incidence of diarrhea in children and adults, especially in those with lower nutritional status (26–32). A recent publication reviewed the use of LR in children with acute diarrhea (1). Of the 14 studies listed, only five addressed the duration of diarrhea/hospitalization in healthy children with AGE. None of the participants were malnourished or received antibiotics. Of these, four studies demonstrated a reduction in duration of diarrhea, while on study found no effect on duration but a reduction in the length of hospitalization. The results in a fifth study did not reach statistical significance but indicated a trend towards reduced severity and symptom duration.

A 2021 systematic review and meta-analysis of probiotics for the treatment of AGE amongst Indian children was conducted by McFarland and colleagues (23). The researchers examined 22 RCTs (*N* = 4,059 participants) including five single-strain probiotics and 3 multi-strained mixtures. For the meta-analyses, 17 RCTs (20 treatment arms) were included. SB had the strongest effect on shortening the duration of diarrhea (standardized mean difference [SMD], −1.86 days; 95% confidence interval [CI], −2.8 to −0.9 days; *P*,0.001). Significant shortening was also seen after LGG (SMD, −1.75 day; 95% CI, −2.73 to −0.77 d; *P* = 0.001) and a mixture of four *Bacillus clausii* strains (O/C, SIN, N/R, T; SMD, −1.39; 95% CI, −2.74 to −0.04; *P* = 0.04). Further, SB and LGG both significantly reduced the duration of hospitalization by SMD −1.81 days (95% CI, −3.58 to −0.04 days; *P* = 0.04) and 1.13 days (95% CI, −2.14 to −0.11 days; *P* = 0.03) respectively. While *B. clausi* proved beneficial for diarrhea in studies conducted in India, it had no effect on duration of hospitalization (SMD, −1.14; 95% CI, −2.91–0.64 days; *P* = 0.21).

McFarland et al. also found that the frequency of stools per day were significant reduced by Day 4 after SB administration and by Day 5 following LGG (23). The authors noted publication bias and significant heterogenicity among studies. It is concluded that in India, two types of probiotics, SB and LGG added to standard treatment were safe and significantly shortened both the duration of diarrhea and hospitalization in children with AGE.

Rotavirus (RV) is the most frequent cause of AGE in non-vaccinated children. As discussed above, probiotics can have an antiviral effect. LGG, for example, acts by inhibiting both cytotoxic and enterotoxic pathogenic mechanisms. Buccigrossi et al. concluded that LGG counteracts RV-induced ion secretion and enterocyte damage by inhibiting oxidative stress and apoptosis by means of specific effects induced by living and postbiotic preparations (52). A 2022 review examined the efficacy of probiotics as antiviral agents for the treatment of RV gastrointestinal infections (24). The authors identified 19 studies exhibiting a statistically significant antiviral effect. Among those tested, SB and LGG emerged as producing the greatest benefit along with various multi-strain probiotics. Their mechanism of action on diarrhea in children is generally agreed to be by immune enhancement and modulation of intestinal microbiota. However, several probiotic studies did not find a significant difference even though well-known strains were used. These findings emphasize the importance of correct dosage, treatment duration and product quality. It should be noted that other factors may affect the efficacy of probiotics. These include the matrix, in which the probiotic is administered. Also, carrier components such as proteins, carbohydrates, and flavoring agents can have a detrimental impact on the quality of probiotic products and their viability (53).

It is worth emphasizing that LGG, SB and LR are not the only probiotics undergoing investigation in RV-related AGE. In a Bangladeshi RCT, 230 male children aged 4–24 months with acute watery diarrhea <2 days’ duration were fed 10^10^ colony-forming units of lyophilized *Lactobacillus paracasei* strain ST11 or placebo daily for 5 days (54). ST11 was found to be ineffective in children with severe RV diarrhea. By comparison, the probiotic was of significant benefit in non-RV diarrhea with reductions in cumulative stool output (ST11 225 +/− 218 vs. Placebo 381 +/− 240 ml/kg), stool frequency (27.9 +/− 17 vs. 42.5 +/− 26), and oral rehydration solution intake (180 +/− 207 vs. 331 +/− 236 ml/kg). A significantly higher proportion of non-RV children receiving ST11 were diarrhea-free within 6 days of treatment (ST11: 76% vs. Placebo: 49%).

A further consideration is safety. Muraro et al. have demonstrated that LGG is safe and well tolerated by children with a cow's milk protein allergy (55).

### Combination of probiotics

Variable effects of administering a single probiotic in patients affected by AGE has led to interest in the potential clinical benefits of combining various bacterial strains. An Indian RCT examined the effects of simultaneous administration of two probiotics, *SB* and *Baccilus subtilis* CU-1 in 180 children aged 6-months to 5-years with mild to moderate acute watery diarrhea (33). The mean duration of diarrhea in the probiotic and placebo groups were similar (54.16 vs. 59.48 h). However, early administration of the combination treatment after onset of diarrhea resulted in significant shortening of symptoms compared to placebo treatment (within 24-hours: 25.21 h; within 48 h: 13.84 h, *p* < 0.05). There were no significant differences in the stool frequencies between the two arms. Similarly, the probiotic combination reduced recurrence of diarrhea and its intensity in the subsequent 3-months.

If a combination of two probiotics can shorten diarrhea, might a cocktail of different ingredients do better? A multi probiotic formula based on the original work by Claudio De Simone is available commercially. The product widely known as VSL#3 consists of eight bacterial strains. A RCT was conducted by Dubey et al. to evaluate efficacy and tolerability of the mixture in the treatment of acute RV-positive diarrhea in children (34). Participants were randomized to receive 4-days of oral mixture or placebo plus usual care. The study was completed by 224 of 230 children (mixture: 113; placebo: 111). Both groups were comparable at entry. On Day 2, a statistically significant lower mean stool frequency and improved stool consistency were found in the mixture group. A reduced ORS requirement was considered to reflect decreased stool volume. These differential benefits persisted until Day 4. No side effects were noted with the use of the probiotic mixture.

Information on the bacterial strains present in many probiotic preparations and their genomes is generally limited. Douillard et al. have undertaken a comparative genomic evaluation of the bacterial strains present in VSL#3 mixture to better understand the product's modes of action (56). Four strains of *Lactobacillus* (*L. acidophilus*, *L. plantarum*, *L.caseii*, and *L.delbrueckii* subspecies *bulgaricus*), three strains of *Bifidobacterium* (*B. breve, B. longum*, and B.*infantis*), and one strain of *Streptococcus* (*S. salivarius* subspecies *thermophilus*) were analyzed. The researchers suggest that the gene clusters of *S. thermophilus* are linked to an effect on the defense system, while *Bifidobacterium* promotes intestinal barrier integrity. Lastly, the genomes of *Lactobacillus* are predicted to encode signaling proteins.

### Prebiotics

In contrast to probiotics, prebiotics are substrates which can be selectively utilized by host microorganisms to confer a health benefit (57). Prebiotics typically consist of non-starch polysaccharides and oligosaccharides. Commonly known prebiotics are oligofructose (fructo-oligosaccharide, FOS), inulin, galacto-oligosaccharides (GOS), lactulose and breast milk oligosaccharides (human milk oligosaccharides or HMOs). The most important groups in terms of their effect on human health are HMOs followed by FOS and GOS. Once ingested, they are degraded by gut microbiota, resulting in short-chain fatty acids, which can affect not only the gastrointestinal tract, but also distant organs (58).

Studies that involve FOS or GOS for treatment of AGE often include other ingredients. For example, in an animal study involving rotavirus-induced diarrhea in suckling rats, a mixture GOS, FOS, pectin-derived acidic oligosaccharides, and heat-treated probiotics in fermented milk components were associated with decreased viral shedding and reduced clinical signs (59). Similarly in humans, an Italian study looking at 120 children enrolled on the first day of diarrheal symptoms, compared locally available hypotonic ORS (225 mOsm/L) to a “superhypotonic’’ ORS (200 mOsm/L) containing Zn, FOS, and xylooligosaccharides (35). The group receiving ORS containing Zn and additional saccharides experienced a 25% higher rate of symptom resolution at 72 h.

### Synbiotics

Synbiotics are a combination of probiotics and prebiotic, which then act together to produce a synergistic effect (57). In one meta-analysis, synbiotics appear to be more effective at reducing the duration of diarrhea and hospitalization than probiotics alone (36). Gonzalez-Ochoa et al. summarized the beneficial effects of pre and probiotics in RV diarrhea (37). One of the main factors responsible for RV pathogenesis is the associated NSP4 protein, a viral toxin able to trigger several cellular responses leading to diarrhea. The RV protein, NSP1, has been linked to the inhibition of interferon production by means of a negative effect on interferon regulatory factors. Probiotics such as *Bifidobacterium* and *Lactobacillus* species in combination with prebiotics such as inulin, Human Milk Oligosaccharides, GOS, and FOS, may lead to a general improvement in the antiviral response and a specific anti-RV effect. Contributory factors include a reduction in RV infectivity and viral shedding, decreased expression of NSP4, and increased levels of specific anti-RV IgAs (22, 37). Clinically, the addition of synbiotics to the low osmolar ORS has been shown to shorten the duration and severity of acute diarrhea by 1–2 days compared to a control group (38). A further benefit in this study was that children with presumed RV-associated diarrhea required no additional medication after commencing on food supplements fortified with synbiotics. Another positive example comes from an emergency room trial conducted in Luxembourg for children with AGE. Here the researchers compared the efficacy of ORS plus a synbiotic food supplement containing several probiotics and FOS vs. a placebo treatment. The consistent finding of more rapid normalization of stool consistency among the actively treated group, supports the findings of earlier research in a primary healthcare setting (39). These findings must, however, be viewed against the recent ESPGHAN position paper which failed to make a definite recommendation in favor of or against synbiotics, for the treatment of a range of gastrointestinal disorders (40).

### Other additives to ORS

#### Several other additives to the ORS formulation have been investigated

Addition of recombinant human lactoferrin and lysozyme to rice-based ORS, were studied by Zavaleta et al. in 140 Peruvian children aged 5–33 months (60). Addition of lactoferrin and lysozyme resulted in a shorter diarrhea duration (3.7 vs. 5.2 days, *P* = 0.05), but not a reduced fecal volume, although in the lactoferrin/lysozyme group there was a greater percentage of children with solid stools in the first 48 h (85 vs. 69%, *P* = 0.04). A meta-analysis of seven clinical trials showed that the addition of glycine failed to reduce either stool output or diarrhea duration in patients with acute non-cholera diarrhea (61). Similarly, Bhan et al. reviewed clinical trials that compared the WHO-ORS and modified ORS to which alanine, glutamine, or maltodextrin had been added; no additional clinical benefit was evident with the modified solution (7). A more recent RCT among 147 dehydrated children in a Mexican hospital also found no benefit of adding L-glutamine compared to standard ORS (14).

## Discussion

A visit to the doctor 3-days after the onset of childhood AGE is a common presentation. Currently, most primary care physicians have little else to offer the distressed child and anxious parents other than reassurance and advice regarding supportive care. In countries where AGE no longer poses a mortality risk, an ORS that aids recovery could improve HRQoL. In addition, reducing the duration of diarrhea could decrease direct and indirect costs of such illnesses. In that regard, several ingredients have been tested. When considering modifications to the standard ORS, three things are important: safety, effectiveness, and cost. Based on multiple trials in infants and young children, the administration of LGG has been shown to be safe (55), while in the developing world, Zn has been shown to be effective in AGE (62). Despite studies demonstrating no significant difference between LGG and placebo (63), an updated review recently confirmed the value of LGG in reducing both the duration of diarrhea and hospitalization (64). The World Gastroenterology Organization Global Guideline “Probiotics and Prebiotics. 2023” also recommends LGG for prevention and reducing duration of diarrhea, length of hospitalization and stool output as well as for some other types of diarrheas (57).

The question is sometimes posed as to whether shortening the duration of AGE-associated diarrhea by 1-day by incorporating LGG in the management, is worthwhile. AGE places a substantial economic burden on the families of affected children. Vandenplas concluded, that while the addition of symbiotics increased initial costs, it greatly reduces the overall burden on the health system, by reducing the need for drugs such as antipyretics, antiemetics, antibiotic, and an antidiarrheal agent (65).

Research in this area is challenging and it may never be possible to reach clear conclusions. The results of this review suggest that a combination of ingredients, i.e., modern reduced osmolarity ORS, plus a prebiotic, probiotic or symbiotic, with Zn and an agent to aid absorption, will benefit some children and adults with AGE. A further consideration is that having something to offer anxious parents and a sick child which is unlikely to harm and may hasten recovery, is a helpful tool for the attending physician while waiting for an anticipated recovery.
